# IL1β Promotes TMPRSS2 Expression and SARS-CoV-2 Cell Entry Through the p38 MAPK-GATA2 Axis

**DOI:** 10.3389/fimmu.2021.781352

**Published:** 2021-12-07

**Authors:** Chiara Cioccarelli, Ricardo Sánchez-Rodríguez, Roberta Angioni, Francisca C. Venegas, Nicole Bertoldi, Fabio Munari, Annamaria Cattelan, Barbara Molon, Antonella Viola

**Affiliations:** ^1^ Department of Biomedical Sciences, University of Padova, Padova, Italy; ^2^ Istituto di Ricerca Pediatrica (IRP), Fondazione Città della Speranza, Padova, Italy; ^3^ Infectious Disease Unit, Padova University Hospital, Padova, Italy

**Keywords:** GATA2, SARS-CoV-2, IL1β, p38, TMPRSS2

## Abstract

After the outburst of the SARS-CoV-2 pandemic, a worldwide research effort has led to the uncovering of many aspects of the COVID-19, among which we can count the outstanding role played by inflammatory cytokine milieu in the disease progression. Despite that, molecular mechanisms that regulate SARS-CoV-2 pathogenesis are still almost unidentified. In this study, we investigated whether the pro-inflammatory milieu of the host affects the susceptibility of SARS-CoV-2 infection by modulating *ACE2* and *TMPRSS2* expression. Our results indicated that the host inflammatory milieu favors SARS-CoV-2 infection by directly increasing TMPRSS2 expression. We unveiled the molecular mechanism that regulates this process and that can be therapeutically advantageously targeted.

## Introduction

At more than one year after the Coronavirus disease 19 (COVID-19) has been declared a global pandemic, the Severe Acute Respiratory Syndrome Coronavirus 2 (SARS-CoV-2) infection is still threatening the healthcare systems around the world. The critical and severe forms of COVID-19 are associated with pneumonia and Acute Respiratory Distress Syndrome (ARDS), conditions that are triggered by deregulated immunity and uncontrolled inflammation. In particular, the inflammatory cytokine milieu of the patient plays a crucial role in COVID-19 progression. Indeed, specific cytokines have been linked to worse severity, longer hospitalization time, or to patient’s age (1). Accordingly, multiple diseases such as diabetes and obesity, as well as aging, that are characterized by chronic inflammation and elevated levels of pro-inflammatory cytokines, represent high risk factors for developing a severe illness after SARS-CoV-2 infection (2). Among the cytokines that have been associated to COVID-19 severity, several reports identified higher levels of IL1β in severe and critical patients compared with mild and moderate ones (3). Actually, SARS-CoV-2 infection is known to trigger the NLRP3 inflammasome activation in various cell types and the activation of this pathway, that leads to a strong increase in IL1β release, might exacerbate ARDS and systemic inflammation (4).

SARS-CoV-2 entry into the target cells involves the binding of the viral spike protein (S) to the host Angiotensin I Converting Enzyme 2 (ACE2) receptor and its proteolytical activation by proteases. Following the binding of the S1 subunit to ACE2 through its receptor-binding domains (RBD), the host transmembrane protease/serine subfamily 2 (TMPRSS2) cleaves the S protein to facilitate membranes fusion (5). The expression levels and the co-expression of *ACE2* and *TMPRSS2* seem to be key factors in determining the susceptibility of target organs to SARS-CoV-2 infection (6). Recently, it has been proved that *ACE2* expression can be regulated in response to interferon alpha and gamma signaling (7), linking the immune response to the modulation of the cell receptors that mediate SARS-CoV-2 entry. TMPRSS2 is expressed in several tissues including lungs, gastrointestinal tract and kidney. Notably, the *TMPRSS2* gene is upregulated in response to androgenic hormones (8), suggesting that the expression of this protease might be involved in the observed higher severity of COVID-19 in men compared to women.

## Results and Discussion

**Figure 1:**
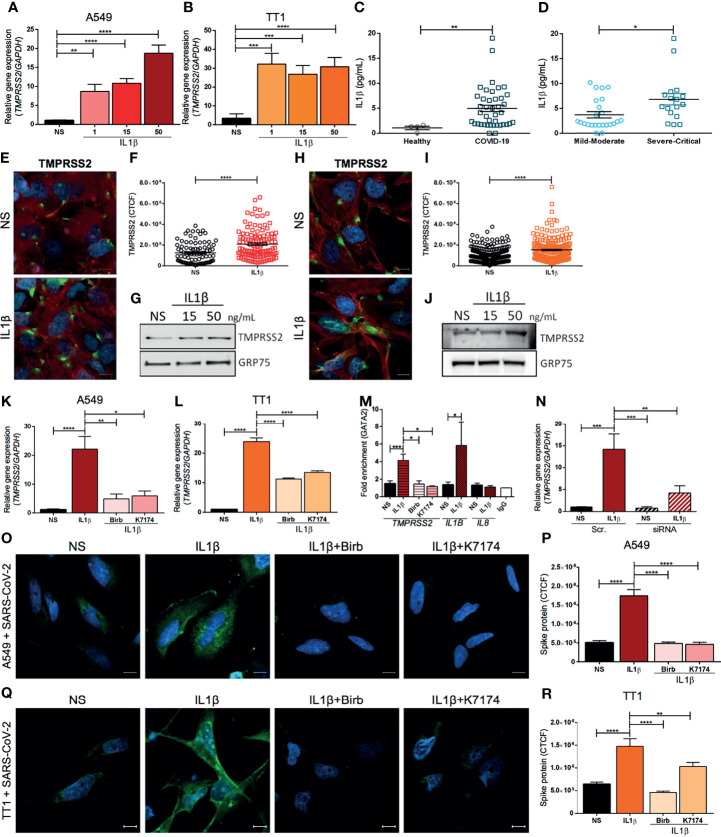
TMPRSS2 expression is up-regulated by inflammatory cytokines enhancing SARS-CoV-2 entry in human lung cells. RT-qPCR analysis of the relative gene expression of *TMPRSS2* in **(A)** A549 and **(B)** TT1 cells either stimulated or not (NS) for 4 hours with IL1β (1, 15 or 50 ng/mL); n=5 independent experiments. IL1β plasma concentration (pg/mL) **(C)** in healthy, age-matched controls and COVID-19 patients, and **(D)** in COVID-19 patients grouped by disease severity (mild-moderate versus severe-critical); n=44 patients. **(E)** Representative immunofluorescence for TMPRSS2 and **(F)** quantification of fluorescence intensity of TMPRSS2 (green) in A549 cell line either stimulated or not (NS) for 24 hrs with IL1β (50 ng/mL). Nuclei were counterstained with Hoechst 33342 (blue) and actin was stained with Phalloidin TexasRed (Red). CTCF: corrected total cell fluorescence. Scale bar: 10 μm; n=3 independent experiments. **(G)** Western blot analysis of TMPRSS2 in total protein lysates from A549 cells stimulated or not (NS) for 24 hours with IL1β (15 or 50 ng/mL); GRP75 was used as a loading control (representative of 3 independent experiments). **(H)** Representative immunofluorescence for TMPRSS2 and **(I)** quantification of fluorescence intensity of TMPRSS2 (green) in TT1 cell line either stimulated or not (NS) for 24 hrs with IL1β (50 ng/mL). Nuclei were counterstained with Hoechst 33342 (blue) and actin was stained with Phalloidin TexasRed (Red). CTCF, corrected total cell fluorescence. Scale bar: 10 μm; n=3 independent experiments. **(J)** Western blot analysis of TMPRSS2 in total protein lysates from TT1 cells stimulated or not (NS) for 24 hours with IL1β (15 or 50 ng/mL); GRP75 was used as a loading control (representative of 3 independent experiments). RT-qPCR analysis of the relative gene expression of *TMPRSS2* in **(K)** A549 or **(L)** TT1 cells stimulated for 4 hours with IL1β (50 ng/mL) and either treated or untreated with Birb796 (p38 MAPK inhibitor, 100 nM) or K7174 (GATA2 inhibitor, 10 µM). NS, Not Stimulated; n=5 independent experiments. **(M)** ChIP-RTqPCR analysis of the fold enrichment of GATA2 binding to the *TMPRSS2* enhancer promotor region, in A549 cells either treated or untreated with Birb796 (p38 MAPK inhibitor, 100 nM) or K7174 (GATA2 inhibitor, 10 µM) and stimulated or not (NS) for 4 hours with IL1β (50 ng/mL). Data were adjusted by input for each sample and normalized with IgG (Mock). The fold enrichment of GATA2 binding to the *IL1B* and to the *IL8* promotors was respectively used as positive and negative control. IgG was used as antibody specificity control. N=3 independent experiments. **(N)** RT-qPCR analysis of the relative gene expression of *TMPRSS2* in A549 cells upon GATA2 silencing with or without stimulation with IL1β (50 ng/mL) for 4 hours. Scramble siRNA (Scr.) was used as a negative control; n=4 independent experiments. **(O)** Representative confocal images and **(P)** quantification of fluorescence intensity of SARS-CoV-2 Spike protein (green) in A549 cells upon infection with heat-inactivated SARS-CoV-2 virus. Cells were stimulated or not (NS) for 8 hours with IL1β (50 ng/mL) and either treated or not with Birb796 (p38 MAPK inhibitor, 100 nM) or K7174 (GATA2 inhibitor, 10 µM). Nuclei were counterstained in blue (Hoechst 33342). Scale bar: 10 μm; n=3 independent experiments. **(Q)** Representative confocal images and **(R)** quantification of fluorescence intensity of SARS-CoV-2 Spike protein (green) in TT1 cells upon infection with heat-inactivated SARS-CoV-2 virus. Cells were stimulated or not (NS) for 8 hours with IL1β (50 ng/mL) and either treated or not with Birb796 (p38 MAPK inhibitor, 100 nM) or K7174 (GATA2 inhibitor, 10 µM). Nuclei were counterstained in blue (Hoechst 33342). Scale bar: 10 μm; n=3 independent experiments. Data are presented as means ± SEM. nonparametric Mann–Whitney U test **(C, D, F, I)** or Kruskal-Wallis test for multiple comparisons with Dunn’s *post hoc* **(A, B, K, L, M, N, P, R)** *P < 0.05, **P < 0.01, ***P < 0.001, ****P < 0.0001.

In this study, we investigated whether the pro-inflammatory milieu of the host affects *TMPRSS2* and *ACE2* expression in SARS-CoV-2 target cells. To this aim, the A549 human lung cell line was exposed to various concentrations of IL1β, IL6, and TNFα inflammatory cytokines and the expression level of *TMPRSS2* and *ACE2* mRNA was evaluated. Interestingly, we found that IL1β and TNFα stimulation enhanced the transcription of *TMPRSS2* (**Figure 1A** and **Supplementary Figure 1A**). On the other side, TNFα and IL1β treatment did not affect *ACE2* expression that was rather increased by IL6 (**Supplementary Figures 1B, C**). The effect of IL1β over *TMPRSS2* and *ACE2* expression was also confirmed in primary immortalized human alveolar epithelial cells (TT1) (**Figure 1B** and **Supplementary Figure 1D**).

In addition to *TMPRSS2*, other proteases, such as *ADAM17* and *FURIN*, have been reported to promote SARS-CoV-2 infection (9) but none of them appeared to be transcriptionally regulated by pro-inflammatory cytokine stimulation in the A549 cells (**Supplementary Figure 2**).

In line with other reports (4), our data confirmed that COVID-19 patients showed an increased level of circulating IL1β (**Figure 1C**) compared to healthy subjects. Notably, IL1β concentration was significantly higher in severe and critical patients compared with mild and moderate ones (**Figure 1D**). Therefore, we focused our analysis on IL1β-induced transcriptional regulation of TMPRSS2 expression. We first validated the induction of TMPRSS2 expression by IL1β stimulation at the protein level by performing immunofluorescence and western blot analyses on both A549 (**Figures 1E–G** and **Supplementary Figure 3**) and TT1 cells (**Figures 1H–J** and **Supplementary Figure 3**). Furthermore, we evaluated whether IL1β stimulation can modulate SARS-CoV-2 endosome and replication related-genes such as IFITM2 (10) and *RNF20* (11). No transcriptional changes were observed for these genes upon IL1β stimulation in lung cells (**Supplementary Figure 4**).

Previous studies showed that *TMPRSS2* expression is controlled by the master regulator transcription factor GATA2 through its binding to the -13 kb DNA enhancer region of the gene (-13 kb upstream the promotor) (8). Furthermore, GATA2 activation has been reported to be mediated by its phosphorylation *via* p38 MAPK pathway (12). GATA2 is also involved in the transcriptional regulation of several pro-inflammatory cytokines, including *IL1B* and *CXCL2* (12). In addition, a positive feedback between IL1β signaling and GATA2 activation has been previously described in Acute Myeloid Leukemia (12). Thus, we speculated that IL1β may induce *TMPRSS2* expression through the p38 MAPK-GATA2 axis. To validate our hypothesis, we first analyzed the effect of IL1β stimulation in A549 and TT1 cells in the presence or absence of specific inhibitors that selectively block p38 MAPK and GATA2 activity. Remarkably, we observed that *TMPRSS2* transcription was significantly decreased by the treatment with p38 MAPK (Birb796) or GATA2 (K7174) inhibitors in IL1β-stimulated A549 and TT1 cells (**Figures 1K, L** and **Supplementary Figure 5A**). *ACE2* transcription was not affected by the inhibitors (**Supplementary Figure 5B**).

In order to confirm the role of GATA2 on the IL1β-induced transcriptional regulation of TMPRSS2, GATA2 chromatin immunoprecipitation was performed. Our results showed that the chromatin enrichment in the TMPRSS2 -13 kb enhancer region was increased upon IL1β stimulation (**Figure 1M**). Importantly, the p38 MAPK and GATA2 inhibitors, that blocked TMPRSS2 overexpression induced by IL1β, were also able to inhibit the GATA2 binding to the enhancer region (**Figure 1M**). Moreover, GATA2 transcription was not affected by pro-inflammatory cytokine exposure (**Supplementary Figure 6**), suggesting that the outlined effect relies on GATA2 activity rather than on a modulation of its expression level. To further corroborate the role of GATA2 on *TMPRSS2* transcription upon IL1β stimulation, we performed GATA2 silencing in A549 cells. Our results confirmed that the downregulation of GATA2 expression blocked the enhancement of TMPRSS2 transcription in response to IL1β exposure. (**Figure 1N** and **Supplementary Figures 7A–C**).

Finally, we evaluated whether the IL1β-induced TMPRSS2 overexpression could increase cell susceptibility to SARS-CoV-2 infection. A549 and TT1 cells were exposed to the non-replicative heat-inactivated SARS-CoV-2 virus, in presence or absence of IL1β and of the selected inhibitors. We observed that IL1β exposure increased the virus entry (**Figures 1O–R** and **Supplementary Figures 7D, E**), which was significantly inhibited by the pharmacological block of the p38 MAPK-GATA2 axis. IL1β has been reported to promote the expression of interferon response genes in epithelial and myeloid cells (13). We found that IL1β stimulation promoted the overexpression of *IRF1* (**Supplementary Figures 8A, B**) and its target (14) *ISG15* (**Supplementary Figures 8C, D**) but not of *IRF9* (**Supplementary Figures 8E, F**) in lung cells. Of note, the expression of these targets has been associated with cytokine storm syndrome in COVID-19 patients (15, 16). Future high throughput studies should be performed to better understand the interconnection between IL1β and innate immune response in SARS-CoV-2 infection.

Collectively, our results indicated that the host inflammatory milieu might favor SARS-CoV-2 infection by increasing TMPRSS2 expression. In addition, we identified the molecular pathway that regulates this process that can be therapeutically targeted. Our findings unveiled a novel pathogenetic mechanism that associates the pro-inflammatory cytokine IL1β with an increased susceptibility to SARS-CoV-2 infection. Importantly, co-downregulation of *IL1B* and *TMPRSS2* upon azithromycin treatment was recently described in basal nasal epithelial cells, further suggesting a link between inflammation and protease expression in SARS-CoV-2 infection (17). Thus, targeting IL1β may not only reduce high inflammatory responses in severe COVID-19 patients, but also limit the viral entry in lung epithelial cells.

## Methods

### Participants, Study Design, and Data Collection

Peripheral Blood (PB) was collected in EDTA tubes from enrolled controls and 44 COVID-19 patients that were admitted to the Infectious and Tropical Disease Unit of the University Hospital of Padua. Plasma was obtained after peripheral blood mononuclear cells (PBMC) isolation by density-gradient sedimentation using Ficoll–Paque PLUS (GE Healthcare, Germany) according to the manufacturer’s protocol. Then, plasma was carefully removed from the 2/3 of the top layer using a sterile serological pipette until the mononuclear cell interphase and stored at −80 °C until the analysis. The median age of the patients was 59.3 years (98-25). All patients were clinically diagnosed with COVID-19 (at least one positive laboratory PCR test for SARS-CoV-2 infection). All patients were classified into mild, moderate, severe, and critical cases based on results from chest imaging, clinical examination, and symptoms (WHO guidelines). The study was performed according to the ethical guidelines of the Declaration of Helsinki (7th revision). The study was approved by the Ethics Committee and the general authorization issued by the Data Protection Authority. Cod CESC n. 4933/AO/20.

### IL1β Quantification

IL1β level was analyzed in the plasma from 4 controls and 44 patients by Luminex assay (Millipore, Billerica, USA). The diluted standard and quality control were used according to the manufacturer’s instructions. The plate was read on Luminex 200™. Analysis was performed using xPONENT 3.1 software.

### Cell Culture and Treatments

Human A549 cell line was donated from ECSIN lab, Padua. The cells were maintained in DMEM high glucose medium supplemented with 1% Na-pyruvate, 1% L-glutamine, 10% Gibco FBS, 1% Penicillin-Streptomycin at 37°C under 5% CO2, while human alveolar epithelial cell line (TT1) were maintained as Montagner et al. reported (18). Cells were stimulated with recombinant human IL1β, IL6 or TNFα (PeproTech) at the concentration of 1, 15 or 50 ng/mL either in combination or not with the inhibitors BIRB 796 (100 nM) or K-7174 (10 µM) (Selleck Chemicals). Vehicle treated and non-stimulated cells were used as a control condition. The treatment medium was composed of DMEM high glucose supplemented with Penicillin-Streptomycin and 2% FBS Gibco. RNA extraction was performed after 4 hours of treatment, whereas protein extraction was performed after 24 hours of treatment.

### Silencing

Cells were transfected with GATA2 Stealth siRNA or scramble Silencer Select Negative Control (Thermo Fisher Scientific), according to the manufacturer’s protocol. Briefly, cells were plated 24h hours before transfection in a growth medium without antibiotics. Stealth siRNA - Lipofectamin2000 (Invitrogen) complexes were prepared according to the manufacturer’s instructions. Both siRNA and scramble were used at the concentration of 75 pmol. 24 hours after transfection cells were stimulated with recombinant human IL1β as previously described. RNA extraction was performed after 4 hours of treatment.

### RNA Extraction, Reverse Transcription, and RT-qPCR

Total RNA was extracted for RT-qPCR analysis from unstimulated and stimulated cells with TRIzol reagent (Thermo Fisher Scientific) following manufacturer instructions. 500 ng of RNA were retrotranscribed using High-Capacity cDNA Reverse Transcription Kit (Thermo Fisher Scientific) according to the manufacturer instructions. cDNA was diluted 1:10 and amplified with specific primers for the human genes *TMPRSS2*, *ACE2*, *GATA2*, *FURIN*, *ADAM17* and *GAPDH* (**Table 1**) using Power SYBR Green Master Mix (Thermo Fisher Scientific) on a QuantStudio™ 5 Real-Time PCR System, 384-well (Applied Biosystems). The analysis of the obtained results was performed by the ΔΔCt method.

**Table 1:** Primers used in this study.

	Forward	Reverse
*GAPDH*	AACAGCCTCAAGATCATCAGC	GGATGATGTTCTGGAGAGCC
*TMPRSS2*	CCTCTAACTGGTGTGATGGCGT	TGCCAGGACTTCCTCTGAGATG
*ACE2*	TCCATTGGTCTTCTGTCACCCG	AGACCATCCACCTCCACTTCTC
*FURIN*	GCCACATGACTACTCCGCAGAT	TACGAGGGTGAACTTGGTCAGC
*ADAM17*	AACAGCGACTGCACGTTGAAGG	CTGTGCAGTAGGACACGCCTTT
-13 kb *TMPRSS2*	GCTGACCTTTAATGAAGTTTG	CCTAGTGAATTTGGCCTCCTC
*IL1B* promoter	TCGCACCCACTTCCTTCTCTT	TGCCAGAGGAAATGGTGACC
*IL8* promoter	GCTGAACCAGAGTTGGAACCC	GGTGCACTGGAGCTGCTTG
*nCoV_IP2*	ATGAGCTTAGTCCTGTTG	CTCCCTTTGTTGTGTTGT
*IRF1*	TTGGCCTTCCACGTCTTG	GAGCTGGGCCATTCACAC
*IRF9*	AACTGCCCACTCTCCACTTG	AGCCTGGACAGCAACTCAG
*ISG15*	GGCTTGAGGCCGTACTCC	CTGTTCTGGCTGACCTTCG
*IFITM2*	ATCCCGGTAACCCGATCAC	CTTCCTGTCCCTAGACTTCAC
*RNF20*	AAAGCATCGCACCATGTCTC	ATCCCACTGCAGGTCATCAA

For the evaluation of cell susceptibility to SARS-CoV-2 infection, concerning the RNA extraction, we followed the protocol by Vogels et al. (19). We then performed reverse transcription as described above and RT-qPCR as described by WHO (20).

### Chromatin Immunoprecipitation

Chromatin immunoprecipitation was performed with MAGnify™ Chromatin Immunoprecipitation System (Thermo Fisher Scientific) according to the manufacturer’s protocol. Briefly, cells were plated and after 24 hours they were starved for 1 hour; then they were stimulated or not with recombinant IL1β (50 ng/mL) and either treated or not with the indicated inhibitors in presence of IL1β (50 ng/mL). After 4 hours of treatment, the DNA-transcription factor crosslink was performed with 1% formaldehyde (Sigma Aldrich) in PBS for 15 min at RT in soft shaking; the reaction was stopped with glycine (Sigma) 125 mM (5 min). Cells were then washed with PBS, scraped in 1,5 mL PBS and resuspended in 100uL of lysis buffer. Cells were then sonicated 20 times at 60 Hz for 20 seconds. To enrich GATA2 DNA binding sites, chromatin was incubated overnight with 1 µg of GATA2 polyclonal antibody (GeneTex, cat#GTX113441). Rabbit IgG was used as an antibody specificity control. DNA was purified using kit provided-magnetic beads. Fold enrichment was calculated following the manufacturer’s instructions after qPCR amplification with Power SYBR Green Master Mix (Thermo Fisher Scientific) on a QuantStudio™ 5 Real-Time PCR System. Reported specific primers for -13kb *TMPRSS2*, *IL1B*, *IL8, nCoV_IP2, IRF1, IRF9, ISG15, IFITM2 and RNF20* were used (**Table 1**) (20–24).

### Protein Extraction and Western Blot

Protein lysates from A549 or TT1 cells were prepared using a lysis buffer (10 mM Tris-HCl pH 9, 4% SDS, 1 mM DTT). 5 µg of total protein extracts were loaded for western blot in Bolt™ 4-12% Bis-Tris Plus gels (Thermo Fisher Scientific) and blotted onto a PVDF membrane (Bio-Rad). Membranes were blocked with 3% bovine serum albumin (Sigma Aldrich) for 1 hour and then incubated with the primary antibodies α-TMPRSS2 antibody (EPR3862, Abcam) or α-GRP75 (D-9, Santa Cruz Biotechnology) at 4°C. After that, membranes were incubated with the secondary antibody goat α-rabbit IgG (H + L)-HRP Conjugate (Bio-Rad) for 1 hour. Chemiluminescence was detected with iBright 1500 (Invitrogen). Images were analyzed with ImageJ software (Fiji) and the protein was quantified and normalized on the housekeeping protein (GRP75) and on the non-stimulated condition.

### Immunofluorescence Analysis

For TMPRSS2 protein immunofluorescence, A549 and TT1 cells were either stimulated or not with IL1β for 24 hours. By contrast, for the evaluation of cell susceptibility to SARS-CoV-2 infection, cells were either stimulated or not with IL1β and the selected inhibitors for 8 hours as previously described. The infection was then performed, using Heat-inactivated SARS-CoV-2 (VR-1986HK, ATCC) at 4 TCID50/mL. 72 hours after the infection, cells were fixed with PFA 4% (Sigma) in PBS, blocked with blocking solution (PBS, 1% BSA, 0,02% NP40) and incubated with SARS-CoV-2 spike antibody (polyclonal, GeneTex) followed by anti-rabbit Alexa Fluor 488 antibody (polyclonal, Invitrogen). Nuclei were counterstained with Hoechst 33342 (Invitrogen) and actin was stained with Phalloidin TexasRed (Invitrogen). Fluorescence images were acquired using the Zeiss LSM 800 confocal laser scanning microscope. The fluorescent signal per cell was quantified using ImageJ software (Fiji) and the corrected total cell fluorescence (CTCF) was calculated. TMPRSS2 staining in A549 and TT1 cells was performed by using H-4 (Santa Cruz Biotechnology) antibody according to the indicated protocol. 3D reconstruction was performed using ZEN 3.2 Blue edition software (Carl Zeiss).

### Statistical Analysis

Data are reported as the mean ± SEM of at least 3 independent experiments. Statistical analysis was performed using GraphPad Prism 6 (GraphPad Software). Comparisons between two groups were performed by the nonparametric Mann–Whitney U test or Kruskal-Wallis test for multiple comparisons with Dunn’s *post hoc* test *P<0.05, **P<0.01, ***P<0.001, ****P<0.0001.

## Data Availability Statement

The raw data supporting the conclusions of this article will be made available by the authors, without undue reservation.

## Ethics Statement

The studies involving human participants were reviewed and approved by the Ethics Committee and the general authorization issued by the Data Protection Authority. Cod CESC n. 4933/AO/20. The patients/participants provided their written informed consent to participate in this study.

## Author Contributions

Conceptualization, AV, RS-R and BM. Investigation CC, FCV, RA, NB, FM, AC and RS-R. Writing- original draft, RS-R, CC and BM. Critical data discussion, AV, BM and RS-R. Funding acquisition AV. All authors contributed to the article and approved the submitted version.

## Funding

The study was funded by Fondazione Città della Speranza, grant number 20/02CoV, and Fondazione Cassa di Risparmio di Padova e Rovigo (CARIPARO, Bando Progetti di Ricerca Covid 2019, n° 55784) to AV.

## Conflict of Interest

The authors declare that the research was conducted in the absence of any commercial or financial relationships that could be construed as a potential conflict of interest.

## Publisher’s Note

All claims expressed in this article are solely those of the authors and do not necessarily represent those of their affiliated organizations, or those of the publisher, the editors and the reviewers. Any product that may be evaluated in this article, or claim that may be made by its manufacturer, is not guaranteed or endorsed by the publisher.

## Supplementary Material

Supplementary Figure 1 Role of IL1β, IL6, and TNFα in SARS-CoV-2 receptors expression. RT-qPCR analysis of the relative gene expression of **(A)** *TMPRSS2* in A549 cells either stimulated or not (NS) for 4 hours with IL6 or TNFα (15 or 50 ng/mL); n=5 independent experiments; **(B)** *ACE2* in A549 cell line either stimulated or not (NS) for 4 hours with IL6 or TNFα (15 or 50 ng/mL); n=3 independent experiments. *ACE2* expression in **(C)** A549 or **(D)** TT1 cells either stimulated or not (NS) for 4 hours with IL1β. Data are presented as means ± SEM. Nonparametric Mann–Whitney U test. ****P < 0.0001, **P < 0.01.

Supplementary Figure 2 ADAM17 and FURIN expression was not transcriptionally modulated by inflammatory cytokines. RT-qPCR analysis of the relative gene expression of **(A)** *ADAM17* and **(B)** *FURIN* in A549 cell line either stimulated or not (NS) for 4 hours with IL1β, IL6 or TNFα (15 or 50 ng/mL); n=3 independent experiments. Data are presented as means ± SEM.

Supplementary Figure 3 TMPRSS2 expression in response to IL1β stimulation in A549 cells. Quantification of western blots for TMPRSS2 protein in **(A)** A549 or **(B)** TT1 cells stimulated or not with IL1β (15 or 50 ng/mL) for 24 hours. Data are presented as means ± SEM. Nonparametric Mann–Whitney U test. *P < 0.05. **(C)** 3D reconstruction of TMPRSS2 (green) immunofluorescent confocal images in A549 and TT1 cells either stimulated or not (NS) with IL1β (50 ng/mL). Nuclei were counterstained with Hoechst 33342 (blue) and actin was stained with Phalloidin TexasRed (Red), n=3 independent experiments.

Supplementary Figure 4 IL1β did not affect transcriptionally endosomal and replication genes for SARS-CoV-2. RT-qPCR analysis of the relative gene expression of **(A, B)** *IFITM2* and **(C, D)** *RNF20* in A549 and TT1 cell line either stimulated or not (NS) for 4 hours with IL1β (50 ng/mL); n=3 independent experiments. Data are presented as means ± SEM.

Supplementary Figure 5 IL1β and selected inhibitors did not affect *ACE2* expression in A549 cell line and the inhibitors vehicle did not alter the observed results. **(A)** RT-qPCR analysis of the relative gene expression of TMPRSS2 in A549 cell line either stimulated or not (NS) with IL1β (50 ng/mL) for 4 hours in presence or not of DMSO (Vehic. of the inhibitors). **(B)** RT-qPCR analysis of the relative gene expression of ACE2 in A549 cell line either stimulated or not (NS) with IL1β (50 ng/mL) and with Birb796 (100 nM) or K7174 (10 µM) for 4 hours, NS: Not Stimulated; n=4 independent experiments. Data are presented as means ± SEM.

Supplementary Figure 6
*GATA2* expression is not affected by inflammatory cytokine stimulation. RT-qPCR analysis of the relative gene expression of GATA2 in **(A)** A549 or **(B)** TT1 cells are either stimulated or not for 4 hours with IL1β (15 or 50 ng/mL); NS: Not Stimulated; n=3 independent experiments. Data are presented as means ± SEM.

Supplementary Figure 7 GATA2 silencing controls and Spike immunofluorescence negative controls. Relative gene expression of **(A)** *GATA2* and **(B)** *E-Cadherin* after GATA2 silencing in A549 cell line. **(C)** Representative western blot for GATA2 and quantification after silencing in A549 cell line. Scramble siRNA was used as a control. n=3 independent experiments. **(D)** Representative confocal images of A549 and TT1 cells without infection with heat-inactivated SARS-CoV-2 virus. **(E)** Relative gene expression of nCoV_IP2 upon infection with heat-inactivated SARS-CoV-2 virus at different time points (2, 24, and 48 hours). Before infection, cells were stimulated or not (NS) for 8 hours with IL1β (50 ng/mL) and either treated or not with Birb796 (p38 MAPK inhibitor, 100 nM) or K7174 (GATA2 inhibitor, 10 µM). Data are presented as means ± SEM. Nonparametric Kruskal-Wallis test for multiple comparisons with Dunn's post hoc. *P < 0.05 **P < 0.01 ***P < 0.001.

Supplementary Figure 8 Effect of IL1β over interferon response genes. Relative gene expression for **(A, B)** *IRF1* and **(C, D)** ISG15 and **(E, F)** *IRF9* in A549 and TT1 cell line either stimulated or not (NS) for 4 hours with IL1β (50 ng/mL); n=3 independent experiments. Data are presented as means ± SEM. Nonparametric Mann–Whitney U test. **P < 0.01, ****P < 0.0001.
