# Supplementary material for: IL1β Promotes TMPRSS2 Expression and SARS-CoV-2 Cell Entry Through the p38 MAPK-GATA2 Axis
Source: Front Immunol. 2021 Dec 7;12:781352. doi: 10.3389/fimmu.2021.781352 (PMC8691651; doi:10.3389/fimmu.2021.781352)

Supplementary Figure 1

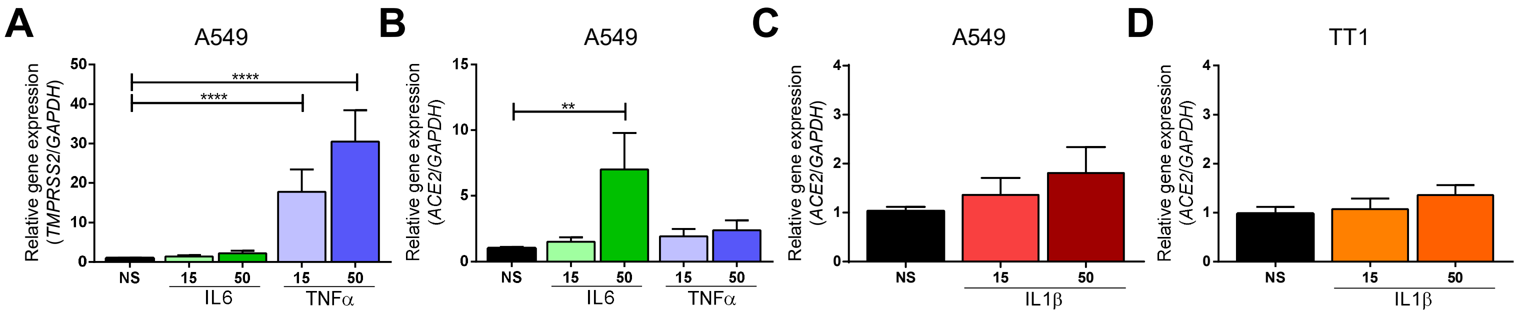

Supplementary Figure 2

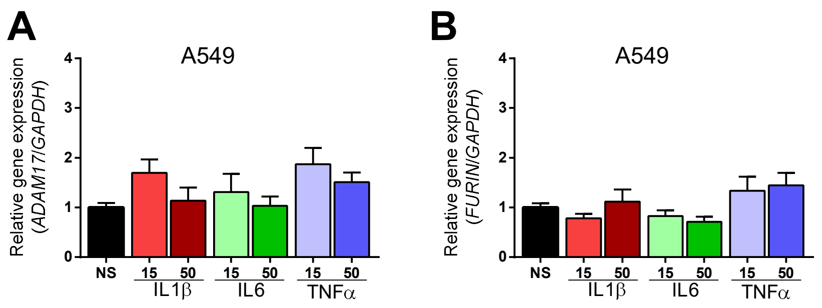

Supplementary Figure 3

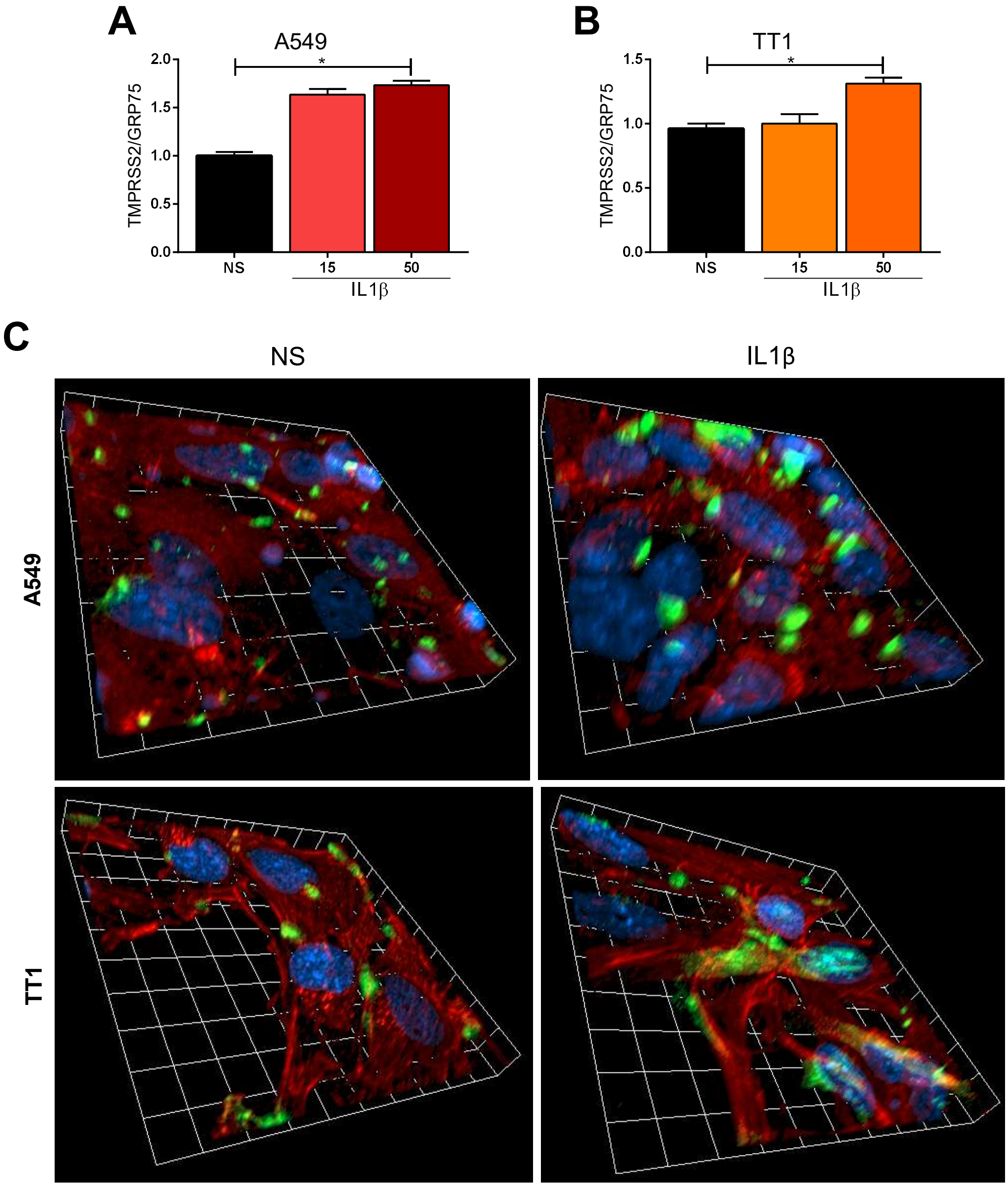

Supplementary Figure 4

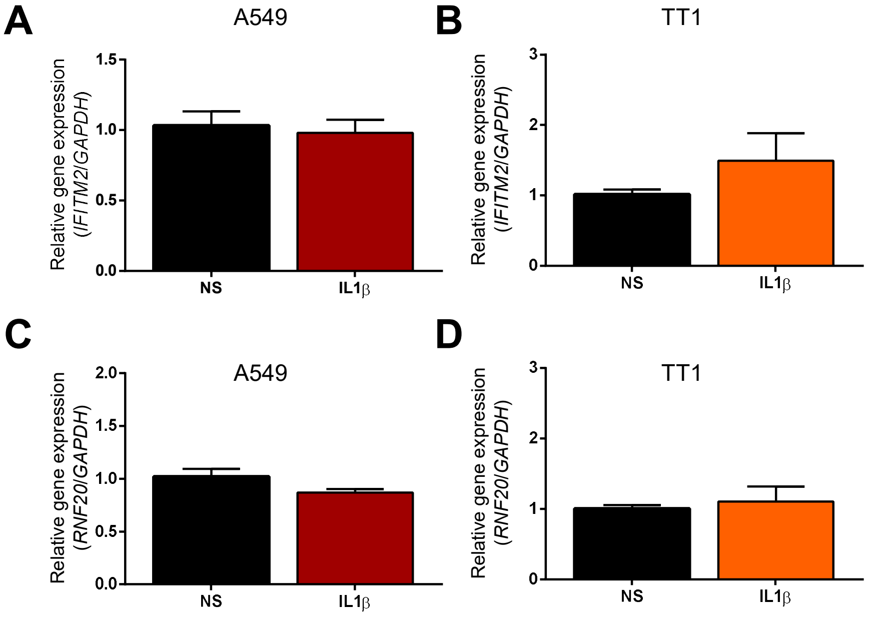

Supplementary Figure 5

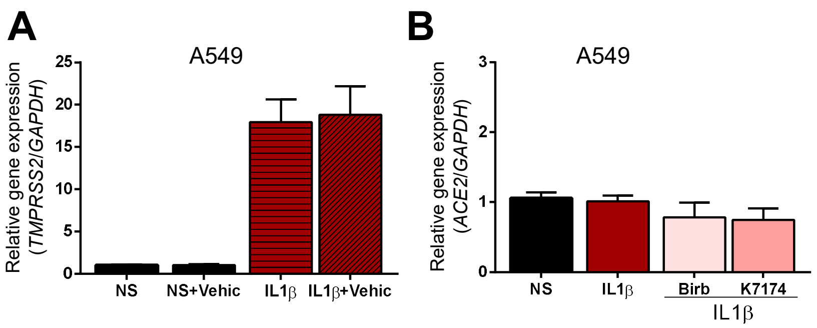

Supplementary Figure 6

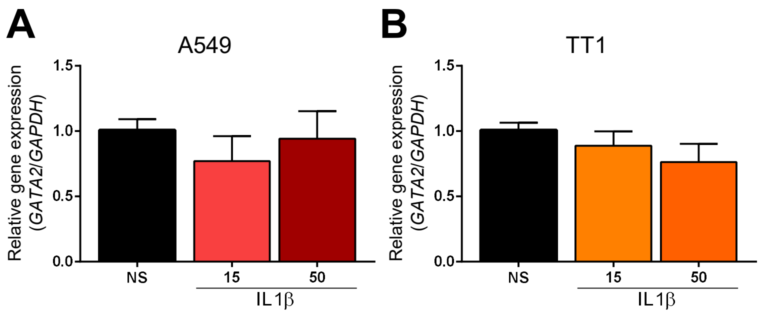

Supplementary Figure 7

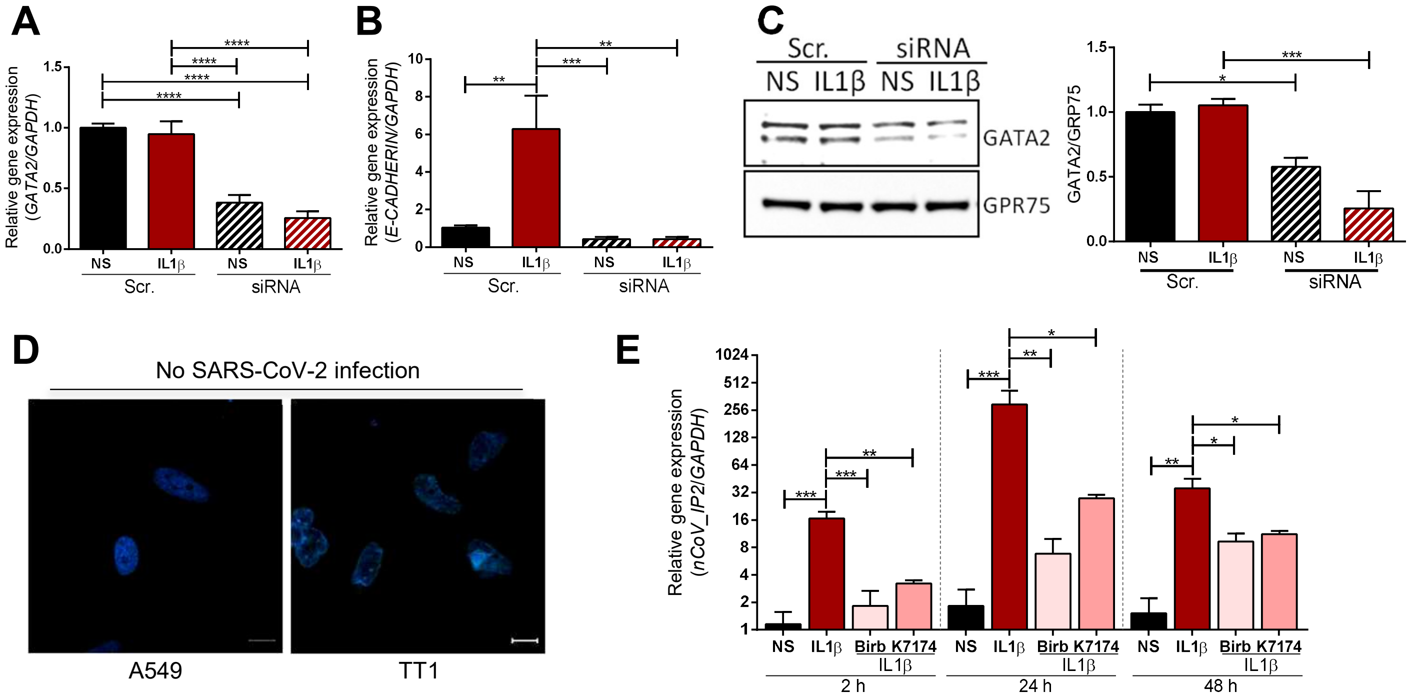

Supplementary Figure 8

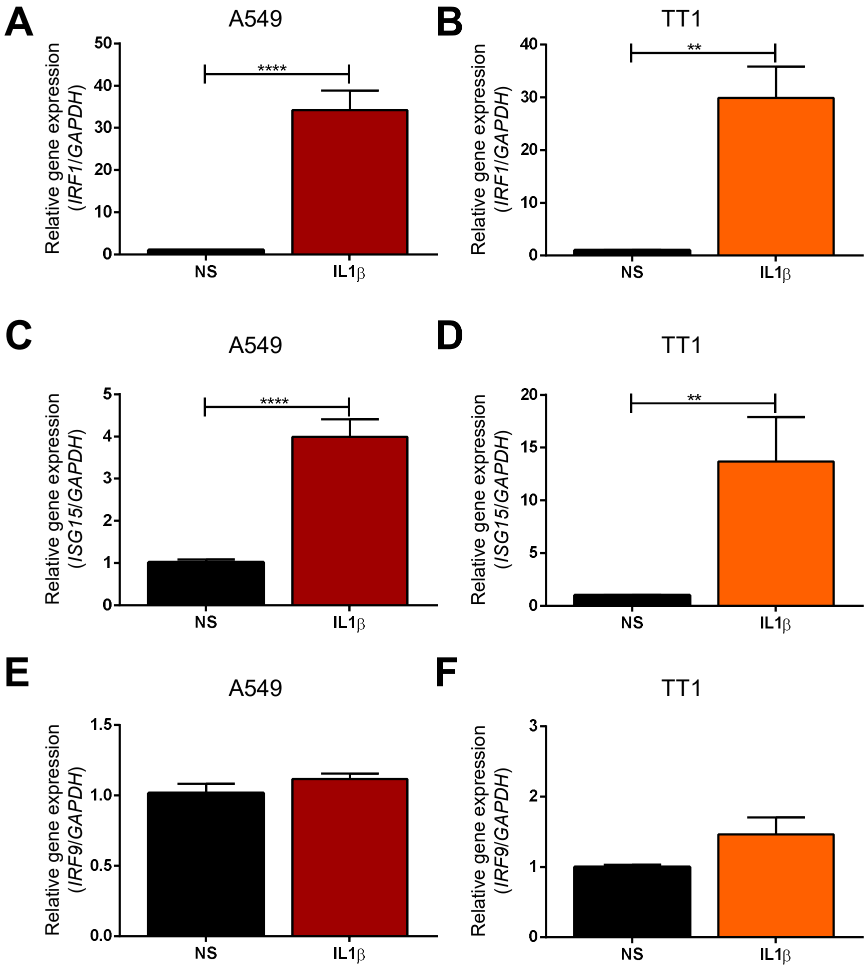

Supplement: Supplementary Figure 1 — Role of IL1β, IL6, and TNFα in SARS-CoV-2 receptors expression. RT-qPCR analysis of the relative gene expression of (A) TMPRSS2 in A549 cells either stimulated or not (NS) for 4 hours with IL6 or TNFα (15 or 50 ng/mL); n=5 independent experiments; (B) ACE2 in A549 cell line either stimulated or not (NS) for 4 hours with IL6 or TNFα (15 or 50 ng/mL); n=3 independent experiments. ACE2 expression in (C) A549 or (D) TT1 cells either stimulated or not (NS) for 4 hours with IL1β. Data are presented as means ± SEM. Nonparametric Mann–Whitney U test. ****P < 0.0001, **P < 0.01. [file DataSheet_1.pdf]
